# Recent Advances in Synthesis, Characterization and Applications of Innovative Materials in Removal of Water Contaminants

**DOI:** 10.3390/ijms24010330

**Published:** 2022-12-25

**Authors:** Thomas Dippong

**Affiliations:** Faculty of Science, Technical University of Cluj-Napoca, 76 Victoriei Street, 430122 Baia Mare, Romania; dippong.thomas@yahoo.ro

Water is a scarce resource with a close and intricate nexus with energy. Water contamination has been reported in almost every region of the world, with a significant impact on human health [1,2]. There are numerous available water decontamination methods, based on various techniques with different efficiencies and operational costs. Engineered nanomaterials with magnetic properties allow the adsorption of contaminants, followed by magnetic separation, while other nanomaterials allow the contaminant’s photodegradation [3,4]. The use of these innovative materials, whether for pollutant adsorption or decomposition by photocatalysis, or for constructing low-cost sensors for the detection of contaminants, has gained interest in the preceding decades.

This Special Issue focuses on the (*i*) application of innovative materials in water decontamination, (*ii*) the synthesis and characterization of engineered nanocomposites, (*iii*) water decontamination by photocatalysis, adsorption and other techniques, and (*iv*) computational and theoretical studies of the reaction mechanisms, kinetics and thermodynamics of water depollution processes.

In this Special Issue, Liao et al. [5] reported the large-scale production and characterization of carbon nanomaterials to remove endocrine-disrupting chemicals (EDCs). EDCs are continuously released and widely spread pollutants in natural environments. At low levels, EDC exposure may cause metabolic, sexual development, and reproductive disorders in aquatic animals and humans [5]. The removal of EDCs from wastewater by adsorption to nanomaterials has wide applicability. Carbon-based nanomaterials (carbon nanotubes, graphene, magnetic carbon nanomaterials, carbon membranes, carbon dots, carbon sponges) have been extensively explored for EDCs adsorption because they are eco-friendly, have good chemical stability, structural diversity, low density, and are suitable for large-scale production [5]. The applications of carbon nanomaterials for the removal of different kinds of EDCs and the adsorption mechanism, as well as recent advances in carbon nanocomposite synthesis and characterization, are discussed [5]. The preparation cost of carbon nanomaterials, such as carbon nanotubes and graphene, is relatively high and there are some technical difficulties with their recyclability [5,6]. It is still challenging to develop new, safe, efficient, and lower-cost carbon nanocomposite adsorbents [5].

Magnetic spinel ferrite (MFe_2_O_4_, where M = Zn, Co, Mn, Ni, etc.) nanoparticles are of high interest to researchers in the fields of materials science and nanotechnology [3,4,6]. The sol–gel route is the most popular means of preparing nanosized ferrites due to its simplicity, low cost, and good control over their structure and properties [3,4,7]. The microwave-assisted sol–gel method combines the advantages of microwave and sol–gel methods, constituting a faster, energy-saving procedure for obtaining single-phase nanopowders of high purity, with accurate control of stoichiometry and capability of industrial scale-up [3,4,7]. Embedding ferrites into a silica (SiO_2_) matrix allows the control of particle growth, minimizes particle agglomeration, and enhances their magnetic guidability and overall biocompatibility [3,4,7].

This Special Issue also includes the study of Dippong et al. [7], an investigation into the structure, morphology and magnetic properties of MFe_2_O_4_ (M = Co, Ni, Zn, Cu, Mn), obtained by thermal decomposition. Unlike similar ferrites embedded in SiO_2_ matrices, single crystalline phases were obtained at both temperatures, excepting the presence of Co_3_O_4_ (CoFe_2_O_4_) and α-Fe_2_O_3_ (MnFe_2_O_4_) at 700 °C [7]. CuFe_2_O_4_ showed the largest particle size (85 nm), while MnFe_2_O_4_ had the smallest particle size (32 nm) [7]. The crystalline CoFe_2_O_4_, heat treated at 1000 °C, displayed the highest saturation magnetization *(M_s_)*, coercive field (*H_C_*) and anisotropy constant *(K*) values, presenting superparamagnetic behavior. Conversely, the other ferrites exhibited paramagnetic behavior [7].

The dependence of structural, morphological and magnetic properties on Ni-Mn substitution in manganese ferrite, synthesized by the sol–gel method and annealed at different temperatures, was reported by Dippong et al. [8]. A number of features were identified, ranging from the presence of poorly crystalline ferrite and highly crystalline mixed cubic spinel ferrite at low annealing temperatures, accompanied by secondary phases at high annealing temperatures, to the gradual decreases in lattice parameters and increases with rising Ni content in the crystallite size, volume, and X-ray density of Mn_1-x_Ni_x_Fe_2_O_4_@SiO_2_ NCs [8]. With increasing Ni content, the saturation magnetization, remanent magnetization, squareness, magnetic moment per formula unit, and anisotropy constant all increase as well, while the coercivity decreases [8]. The magnetic properties of the NCs were strongly dependent on chemical composition, cation distribution between tetrahedral (A) and octahedral (B) sites, as well as on surface effects derived from the synthesis methods [8].

The study of Dippong et al. [9], focusing on the impact of Ni content on the structure and sonophotocatalytic activity of Ni-Zn-Co ferrite nanoparticles, was also included in this Special Issue. The obtained results indicated the formation and decomposition of metal succinate precursors in two stages, with distinct formation and decomposition of divalent (Ni^2+^, Zn^2+^, Co^2+^) and trivalent (Fe^3+^) succinates [9]. The XRD analysis revealed the presence of well-crystallized ferrites along two crystalline phases of the SiO_2_ matrix (cristobalite and tridymite) [7]. In samples with high Zn content, traces of hematite were also identified [9]. Both the agglomeration of particles and the particle size of Ni-Zn-Co ferrites increase with the rising Ni content, the latter growing from 34 nm to 40 nm [9]. All samples showed an excellent optical response in the visible range, the best sonophotocatalytic performance being found for the Ni_0.3_Zn_0.3_Co_0.4_Fe_2_O_4_@SiO_2_ sample, most likely due to the equilibrium between Ni-ferrite and Zn-ferrite [9].

In this Special Issue, the selective recovery of cadmium, cobalt, and nickel from spent Ni–Cd batteries was reported by Weshahy et al., who used adogen^®^ 464 and mesoporous silica derivatives [10]. Spent Ni–Cd batteries are now considered an important source for many valuable metals [10]. Adogen 464 was used for Cd^2+^ extraction, followed by precipitation as a yellow CdS product with 0.5% Na_2_S solution, by setting the pH at 1.25 and maintaining room temperature conditions. The optimum leaching process was achieved using 20% H_2_SO_4_, solid/liquid (S/L) 1/5 at 80 °C for 6 h [10]. The leaching efficiency of Fe, Cd, and Co was nearly 100%, whereas the leaching efficiency of Ni was 95% [10]. The prepared 1,1′-(4-hydroxy-1,3-phenylene) bis(3-(3-(triethoxysilyl)propyl)urea-bridged mesoporous organosilica (PTU-MS) silica was applied for adsorption of Co(II) ions from aqueous solution, while the desorption process was performed using 0.3 M H_2_SO_4_ [10].

The study of Ninciuleanu et al. [11], focusing on the impact of adjusting some properties of poly (methacrylic acid) (nano) composite hydrogels by means of silicon-containing inorganic fillers, was also included in this Special Issue. The effect of these fillers, in correlation with their characteristics, structure and swelling, as well as the viscoelastic and water decontamination properties of (nano)composite hydrogels, were studied comparatively [11]. The experiments demonstrated that the nanocomposite hydrogel morphology was determined by the way the filler particles were dispersed in water [11]. The structure of poly (methacrylic acid) hydrogels was also affected by the reinforcing agent through the pH of its aqueous dispersion [11]. The two Laponite XLS/XLG clays and montmorillonite led to exfoliated and intercalated nanocomposites, respectively, while pyrogenic silica formed agglomerations of spherical nanoparticles within the hydrogel [11]. The equilibrium swelling degree depended on both the pH of the environment and the filler nature [11]. At approximately constant swelling degree, the filler addition improved the mechanical properties of the (nano)composite hydrogels, while after equilibrium swelling (pH 5.4), the viscoelastic moduli values depended on the filler [11]. The rheological measurements also showed that the strongest hydrogels were obtained in the case of the Laponite XLS/XLG clays reinforcing agent [11]. The synthesized (nano)composite hydrogels displayed a different ability to decontaminate cationic dye-containing waters as a function of the filler included, with the highest absorption rate and absorption capacity being displayed by the Laponite XLS-reinforced hydrogel [11]. The (nano)composite hydrogels discussed here may also find applications in the pharmaceutical field as substances to mediate the controlled release of drugs [11].

In this Special Issue, the effective removal of sulfonated pentablock copolymer coating of polypropylene filters for dye and metal ions by integrated adsorption and filtration process was reported by Filice et al. [12]. The polypropylene (PP) fibrous filters, equipped with sulfonated pentablock copolymer (s-PBC) layers, were tested for their capacity to remove cationic organic dyes, such as methylene blue and heavy metal ions (Fe^3+^ and Co^2+^), from water by adsorption and filtration [12]. Polymer-coated filters showed high efficiency in removing methylene blue from an aqueous solution in both the absorption and filtration processes, with 90% and 80% removal rates, respectively [12]. The coated filters showed a better performance removing heavy metal ions (Fe^3+^ and Co^2+^) during filtration than adsorption [12]. In the adsorption process, controlled interaction times allow the ionic species to interact with the surface of the filters leading to the formation and release of new species in solution [12]. During filtration, the ionic species are easily trapped in the filters, especially those that are UV-modified. A total removal (>99%) via a single filtration process was observed for Fe^3+^ ions [12]. The filtration processes are faster, and, therefore, the interaction time is not sufficient to release reaction byproducts into the solution; the ionic species are easily trapped in the filters, in particular the filters whose surface was modified by UV treatment [12]. It has also been shown that the treatment increases the hydrophilicity of the filters, enhancing their filtration capacity [12]. Although further work is needed to extensively investigate the lifetime and regeneration processes of such filters, the functional polymeric coating of commercial and low-cost filters is a promising strategy for the effective removal of pollutants from water [12].

This Special Issue also includes the study of Kim et al. [13], which highlighted the granulation of bismuth oxide by alginate for iodide removal from water. The granulation of bismuth oxide by alginate were presented, along with the iodide adsorption efficacy of alginate–bismuth oxide for different initial iodide concentrations and contact time values [13]. Bismuth oxide appeared in two forms: Bi_2_O_2.33_ and γ-Bi_2_O_3_, and was successfully granulated with alginate, yielding spherical beams with a diameter of 3 mm. The intraparticle pores in the granule could enhance iodide adsorption [13]. Iodide adsorption by alginate–bismuth oxide gradually increased and did not reach a plateau, even at an initial iodide concentration of 1000 mg/L. The process occurred as monolayer adsorption by the chemical interaction and precipitation between bismuth and iodide, followed by physical multilayer adsorption at a very high concentration of iodide in solution [13]. The surface and cross-section after iodide adsorption indicated that the adsorbed iodide interacted with bismuth oxide in alginate–bismuth oxide through Bi–O–I complexation [13]. These data showed that alginate–bismuth oxide is a promising iodide adsorbent with a high absorption capacity and stability, and it can help to prevent secondary pollution [13].

In this Special Issue, Chen et al. reported on the one-step carbonization synthesis of magnetic biochar (BMFH/Fe_3_O_4_) with a 3D network structure, undertaken by controlling different heating conditions in a high-temperature process, as well as its application in organic pollutant control [14]. The microbial filamentous fungus *Trichoderma reesei* was used as a template, and Fe^3+^ was added to the culture process, which resulted in uniform recombination through the bio-assembly properties of fungal hyphae [14]. The presence of magnetic nanoparticles allows researchers to recover biochar from water and convert it into a Fenton-like catalytic reagent that improves treatment efficiency [14]. The catalytic degradation of organic pollutants reached 99% in 60 min [14]. After 10 cycles, malachite and tetracycline hydrochloride removal by BMFH/Fe_3_O_4_ remained above 80% [14].

This Special Issue also includes the study of Liu et al. [15] on a facile and quick preparation of Bi_2_Fe_4_O9/Bi_25_FeO_40_ hetero structures with enhanced photocatalytic activity for the removal of antibiotics by the hydrothermal method, combined with spark plasma sintering (SPS). Bismuth ferrite-based heterojunction composites are promising visible light-responsive photocatalysts because of their narrow band gap structure; however, the synthetic methods reported in the literature were usually time-consuming [15]. It was found that the formation of a well-defined heterojunction between Bi_2_Fe_4_O_9_ and Bi_25_FeO_40_ speeds up the transformation and separation of photoinduced carriers, enhancing their photoelectric properties and photocatalytic performance [15]. The possible influence factors of spark plasma sintering on photoelectric and photocatalytic performance of bismuth ferrite-based composites were also discussed [15]. This study provides a simple, feasible and economical method for the facile and quick synthesis of a highly active bismuth ferrite-based visible light-driven photocatalyst for practical applications [15].

The study of Zhu et al. [16] on the preparation of a Z-type g-C_3_N_4_/(A-R)TiO_2_ composite catalyst and its mechanism for the degradation of gaseous and liquid ammonia is also presented in this Special Issue. The g-C_3_N_4_/(A-R)TiO_2_ composite catalyst had a better dispersion, a smaller band gap width, a larger specific surface area, a stronger light absorption capacity, and a stronger photogenerated carrier separation ability than (A-R)TiO_2_ catalyst [16]. Gaseous and liquid ammonia were used as the target pollutants to investigate the activity of the prepared catalysts, and the results showed that the air wetness and initial concentration of ammonia had a great influence on its degradation [16]. The superoxide anion radical (O_2_^−^) and hydroxyl radical (OH) were the main active components in the photocatalytic reaction process [16]. The photogenerated electrons in the conduction band of (A-R)TiO_2_ catalyst transferred to the valence band of g-C_3_N_4_ and combined with the photogenerated holes in the valence band of g-C_3_N_4_, forming a Z-type heterostructure that significantly improved the efficiency of the photogenerated electron–hole migration and separation, thus increasing the reaction rate [16].

This Special Issue also includes the study of Zheng et al. [17], that reported a size effect in hybrid TiO_2_:Au nanostars as a promising alternative method with which to remove contaminants of emerging concern from wastewaters under sunlight irradiation. TiO_2_:Au nanostars with different Au component sizes and branching were generated and tested in the degradation of ciprofloxacin. They showed the highest photocatalytic degradation, between 83% and 89% under UV and VIS radiation, together with a threshold in photocatalytic activity in the red region [17]. The large size of the Au-branched nanoparticles extended the light absorption to the visible range, in addition to part of the NIR region, and reduced the bandgap from 3.10 eV to 2.86 eV, respectively [17]. The applicability of TiO_2_:Au-NSs with lower branching and optimum performance was further explored with their incorporation into a porous matrix, based on PVDF-HFP. The concept behind this was to open the way for a reusable energy cost-effective system in the photo-degradation of emerging contaminants [17]. The membranes were produced successfully and presented high porosity and a well-distributed porous structure [17].

In this Special Issue, Feng et al. reported that the synergistic effect of adsorption–photocatalysis for the removal of organic pollutants on mesoporous Cu_2_V_2_O_7_/Cu_3_V_2_O_8_/g-C_3_N_4_ heterojunctions (CVCs) enhanced visible light absorption and improved the separation efficiency of photoinduced charge carriers [18]. CVCs exhibited superior adsorption capacity and photocatalytic performance in comparison with pristine g-C_3_N_4_ (CN) CVC-2 (containing 2 wt% of Cu_2_V_2_O_7_/Cu_3_V_2_O_8_) [18]. Good synergistic removal efficiencies were obtained for dyes (96.2% for methylene blue, 97.3% for rhodamine B) and antibiotics (83.0% for ciprofloxacin, 86.0% for tetracycline and 80.5% for oxytetracycline) [18]. The pseudo-first-order rate constants of methylene blue and rhodamine B photocatalytic degradation on CVC-2 were 3 times and 10 times that of pristine g-C_3_N_4_. This work provides a reliable reference for wastewater treatment [18].

This Special Issue also includes the study of Su et al. [19] on the one-step synthesis of nitrogen-doped porous biochar, based on the nitrogen-doping co-activation method and its application in the control of water pollutants. Birch bark (BB) was used for the first time to prepare porous biochars via different one-step methods, including direct activation (BBB) and N-doping co-activation (N-BBB) [19]. The specific surface area and total pore volume of BBB and N-BBB were 2502.3 and 2292.7 m^2^/g, and 1.1389 and 1.0356 cm^3^/g, respectively, proving the feasibility of N-doping co-activation in pore-forming [19]. The large specific surface area and the high total pore volume played a substantial role in the adsorption process. Both BBB and N-BBB showed excellent capacity to remove methyl orange dye and Cr^6+^. The adsorption capacity of N-BBB remained above 80% after five regenerations, which fully proved the stability of regeneration [19]. Moreover, the excellent adsorption performance of N-BBB may have been influenced by pore filling, π–π interaction, H-bond interaction, and electrostatic attraction, all of which supported the biochars’ high performance. This study provided new directions for biomass valorization [19].

In this Special Issue, Piras et al. reported the influence of the defects and morphology of undoped and Al-doped ZnO nanoparticles on the photocatalytic degradation efficiency of Rhodamine B under UV-Visible [20]. The undoped ZnO nanopowder, annealed at 400 °C, resulted in the highest degradation efficiency of ca. 81% after 4 h under green light irradiation (525 nm) in the presence of 5 mg of catalyst [20]. Photoluminescence showed that the insertion of a dopant increases the oxygen vacancies, reducing the peroxide-like species responsible for photocatalysis [20]. The annealing temperature helps to increase the number of red-emitting centers up to 400 °C, while at 550 °C the photocatalytic performance drops due to the aggregation tendency [20]. These results suggest the interconnection between defects, synthesis and post-synthesis routes, particle size and photocatalytic activity [20]. The high amount of defect that is not absorbed in the green region, and the reduction of O_2_^2−^ species, possibly deactivate the photocatalytic process. This has the effect of rendering the Al-doped ZnO catalysts inactive for the photodegradation of the Rhodamine B dye in an aqueous solution under green light [20].

This Special Issue also includes the study of Babilas et al. [21] on the recovery of the N,N-Dibutylimidazolium chloride ionic liquid from aqueous solutions by an electrodialysis method. The fact that the recovery ratio, the [C_4_C_4_IM]Cl molar flux, and the electric current efficiency increase with a rising concentration of [C_4_C_4_IM]Cl in the feed solution was reported [21]. The energy consumption also highly depends on the initial [C_4_C_4_IM]Cl content and increases linearly with the increase in the [C_4_C_4_IM]Cl concentration in the initial solution. The ionic liquids concentration in the feed solution influences the solution conductivity, electrical resistance, and concentration polarization, as well as the electrodialysis efficiency [21] by a reduction in electrical resistance of the initial dilute with the increasing ionic liquids concentration and the acceleration of ions transport across membranes [21].

In this Special Issue, Zheng et al. reported water tree characteristics and its mechanism of crosslinking polyethylene grafted with polar group molecules. [22]. The researchers aimed to restrain the electric stress impacts of water micro-droplets on insulation defects under electric fields in crosslinked polyethylene material. To do so, chemical graft modifications were performed by introducing chloroacetic acid allyl ester and maleic anhydride individually, as two specific polar group molecules, into polyethylene material via a peroxide melting approach [22]. Combined with Monte Carlo molecular simulations, it is verified that water tree resistance can be significantly promoted by grafting polar group molecules [22]. The grafted polar groups can enhance Van der Waals’ forces between polyethylene molecules and are available as heterogeneous nucleation centers for polyethylene crystallization, both of which lead to the increased densities of spherulites with reduced-volume and increased-tenacity amorphous regions between lamellae. These developments account for the considerable improvement in water tree resistance [22]. The crosslinked polyethylene materials improve the insulation performances of polyethylene insulation materials in high-voltage cable manufacturing by grafting on polar group molecules [22].

This Special Issue also includes the study of Liu et al. [23] on the sensitivity of plastic nanoparticle transportation to typical phosphates associated with ionic strength and solution pH. The influence of phosphates on the transport of plastic particles in porous media is environmentally relevant due to their ubiquitous coexistence in the environment [23]. The trends of plastic nanoparticles transport vary with increasing concentrations of NaH_2_PO_4_ and Na_2_HPO_4_ due to the coupled effects of increased electrostatic repulsion, the competition for retention sites, and the double layer compression [23]. Hydrogen bonding from two phosphates that act as proton donors contributes to variations in the interactions of plastic nanoparticles and porous media and thus influences plastic nanoparticles transport [23]. High pH values tend to increase the rate of plastic nanoparticles transportation due to their enhanced deprotonation of surfaces [23]. The presence of physicochemical heterogeneities on solid surfaces can reduce rates of plastic nanoparticles transport and increase the sensitivity of the transport to ionic strength [23]. This study highlights the sensitivity of plastic nanoparticles transport to phosphates and contributes to the better understanding of the fate of plastic nanoparticles and other colloidal contaminants in the environment [23].

In this Special Issue, Yu et al. reported the impact of acetate on the reduction of perchlorate by mixed microbial culture under the influence of nitrate and sulfate, the kinetic parameters of the Monod equation and the optimal ratio of acetate to perchlorate for the perchlorate-reducing bacterial consortium. [24]. The biological reduction of contaminants such as perchlorate (ClO_4_^−^) is considered to be a promising water treatment technology: the process is based on the ability of a specific mixed microbial culture to use perchlorate as an electron acceptor in the absence of oxygen. Both fixed optimal hydraulic retention times and the effect of nitrate on perchlorate reduction were investigated with various concentrations of the electron donor [24]. The presence of sulfate in wastewater did not affect the perchlorate reduction. However, perchlorate reduction was inhibited in the presence of nitrate during exposure to a mixed microbial culture [24].

This Special Issue also includes the study of Ran et al. [25] on the removal of methylene blue by EuVO_4_/g-C_3_N_4_ mesoporous nanosheets via coupling adsorption and photocatalysis. The ultrathin and porous structure of the EuVO_4_/g-C_3_N_4_ increased the specific surface area and active reaction sites, and the formation of the heterostructure extended visible light absorption and accelerated the separation of charge carriers [25]. These two factors were advantageous in promoting the synergistic effect of adsorption and photocatalysis, and ultimately enhanced the methylene blue’s adsorption capability and photocatalytic removal efficiency [25]. The methylene blue adsorption on EuVO_4_/g-C_3_N_4_ followed the pseudo second-order kinetics model, and the adsorption isotherm data complied with the Langmuir isotherm model [25]. The photocatalytic degradation data of methylene blue on EuVO_4_/g-C_3_N_4_ obeyed the zero-order kinetics equation for 0–10 min and the first-order kinetics equation for 10–30 min [25]. This study provided promising EuVO_4_/g-C_3_N_4_ heterojunctions, with superior synergetic effects of adsorption and photocatalysis, for potential application in wastewater treatment [25].

The study of Iosif et al. [26] is focused on the orthodontic bracket’s interaction with an acidic environment, induced by regular consumption of soft drinks and energy drinks, and the enamel quality after debonding. It was found that the adhesive layer has the most important role in properly binding the brackets to the enamel surface [26]. Two categories of adhesives were investigated: resin-modified glass ionomer and resin composite adhesives [26]. It was observed that the resin-modified glass ionomer is more resistant in an acidic environment, assuring a better bond strength than the resin composite because of better insulation of the filler particles, preventing their direct contact with acids in soft drinks [26]. The SEM and AFM complex investigations of the enamel surface, after bracket debonding and exposure to acidic environments, reveal that it is more affected by soft drinks containing phosphoric acid after resin composite debonding and by energy drinks with citric acid content after resin-modified glass ionomer debonding [26]. Additionally, it was found that proper polishing of the enamel surface after debonding assures good protection against further erosion [26].

I am grateful to all the authors for their contributions, covering the most recent progress and new developments in the field of metal–ferrite nanocomposites. I hope that the published studies will pave the way for novel real-world synthesis, characterization, and applications of innovative materials.
